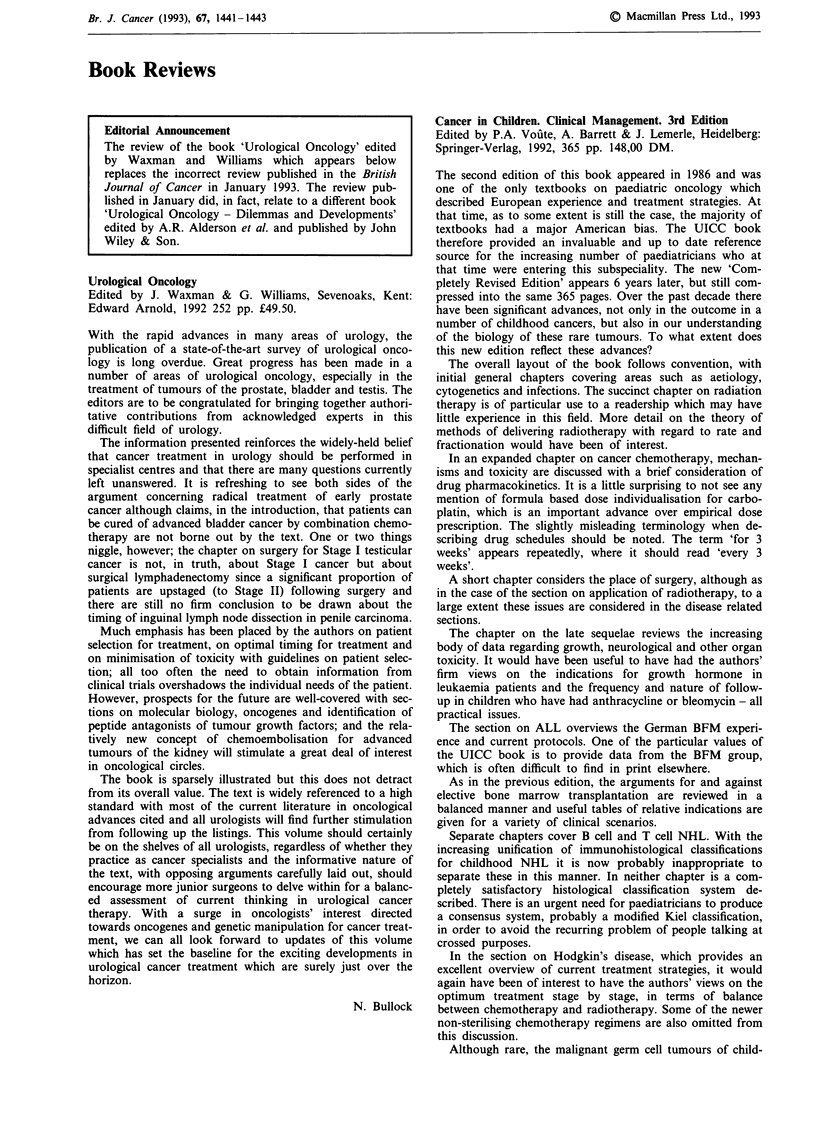# Urological Oncology

**Published:** 1993-06

**Authors:** N. Bullock


### Book Reviews

**Editorial Announcement**

The review of the book 'Urological Oncology' edited by Waxman and Williams which appears below replaces the incorrect review published in the *British Journal of Cancer* in January 1993. The review published in January did, in fact, relate to a different book 'Urological Oncology – Dilemmas and Developments' edited by A.R. Alderson *et al.* and published by John Wiley & Son.

**Urological Oncology**

Edited by J. Waxman & G. Williams, Sevenoaks, Kent: Edward Arnold, 1992 252 pp. £49.50.

With the rapid advances in many areas of urology, the publication of a state-of-the-art survey of urological oncology is long overdue. Great progress has been made in a number of areas of urological oncology, especially in the treatment of tumours of the prostate, bladder and testis. The editors are to be congratulated for bringing together authoritative contributions from acknowledged experts in this difficult field of urology.

The information presented reinforces the widely-held belief that cancer treatment in urology should be performed in specialist centres and that there are many questions currently left unanswered. It is refreshing to see both sides of the argument concerning radical treatment of early prostate cancer although claims, in the introduction, that patients can be cured of advanced bladder cancer by combination chemotherapy are not borne out by the text. One or two things niggle, however; the chapter on surgery for Stage I testicular cancer is not, in truth, about Stage I cancer but about surgical lymphadenectomy since a significant proportion of patients are upstaged (to Stage II) following surgery and there are still no firm conclusion to be drawn about the timing of inguinal lymph node dissection in penile carcinoma.

Much emphasis has been placed by the authors on patient selection for treatment, on optimal timing for treatment and on minimisation of toxicity with guidelines on patient selection; all too often the need to obtain information from clinical trials overshadows the individual needs of the patient. However, prospects for the future are well-covered with sections on molecular biology, oncogenes and identification of peptide antagonists of tumour growth factors; and the relatively new concept of chemoembolisation for advanced tumours of the kidney will stimulate a great deal of interest in oncological circles.

The book is sparsely illustrated but this does not detract from its overall value. The text is widely referenced to a high standard with most of the current literature in oncological advances cited and all urologists will find further stimulation from following up the listings. This volume should certainly be on the shelves of all urologists, regardless of whether they practice as cancer specialists and the informative nature of the text, with opposing arguments carefully laid out, should encourage more junior surgeons to delve within for a balanced assessment of current thinking in urological cancer therapy. With a surge in oncologists' interest directed towards oncogenes and genetic manipulation for cancer treatment, we can all look forward to updates of this volume which has set the baseline for the exciting developments in urological cancer treatment which are surely just over the horizon.

N. Bullock